# A rare case of nephroblastoma arising in a multicystic dysplastic kidney: a case report and review of the literature

**DOI:** 10.1093/jscr/rjaf003

**Published:** 2025-01-31

**Authors:** Sabrine Ben Youssef, Rim Dghaies, Afef Toumi, Manel Njima, Randa Salem, Myriam Ben Fredj, Nouha Ammar, Imene Chabchoub, Amine Ksia, Lassaad Sahnoun

**Affiliations:** Pediatric Surgery Department, Fattouma Bourguiba University Hospital, Street of June 1, 1995 - Monastir - 5000, Tunisia; Pediatric Surgery Department, Fattouma Bourguiba University Hospital, Street of June 1, 1995 - Monastir - 5000, Tunisia; Pediatric Surgery Department, Fattouma Bourguiba University Hospital, Street of June 1, 1995 - Monastir - 5000, Tunisia; Anatomopathology Department, Fattouma Bourguiba University Hospital, Street of June 1, 1995 - Monastir - 5000, Tunisia; Radiology Department, Fattouma Bourguiba University Hospital, Street of June 1, 1995 - Monastir - 5000, Tunisia; Pediatric Surgery Department, Fattouma Bourguiba University Hospital, Street of June 1, 1995 - Monastir - 5000, Tunisia; Oncology Department, Farhat Hached University Hospital, Ibn El Jazzar Street, Sousse, Tunisia; Oncology Department, Farhat Hached University Hospital, Ibn El Jazzar Street, Sousse, Tunisia; Pediatric Surgery Department, Fattouma Bourguiba University Hospital, Street of June 1, 1995 - Monastir - 5000, Tunisia; Pediatric Surgery Department, Fattouma Bourguiba University Hospital, Street of June 1, 1995 - Monastir - 5000, Tunisia

**Keywords:** multicystic dysplastic kidney, Wilms’ tumor, children

## Abstract

The simultaneous occurrence of Wilms tumor (WT) and multicystic kidney disease (MCKD) is extremely uncommon. Diagnosing WT in pediatric patients with multicystic dysplastic kidney (MCDK) substantially impacts management strategies, especially in surgical interventions and long-term outcomes. In summary, while the exact prevalence of WT in children with MCKD is not well-defined, children with MCDK are followed up throughout childhood by ultrasound because of the perceived risk of developing WT, although this risk is poorly defined and somewhat controversial. Herein, we present the case of an 8-year-old child diagnosed with WT arising in a clearly defined MCDK, discovered incidentally through histological analysis. This case contributes to the ongoing discussion by adding to the existing reports in the literature.

## Introduction

Wilms tumor (WT), also known as nephroblastoma, is a type of pediatric kidney cancer, representing 5% of all childhood cancers and 95% of renal cancers in children [1]. The average age of onset is 3 years for sporadic cases and 2 years for hereditary cases [2]. WT is believed to arise from poorly differentiated mesenchymal renal stem cells [3]. Multicystic dysplastic kidney (MCDK), on the other hand, is a developmental renal anomaly characterized by the replacement of renal parenchyma with non-communicating cysts and atresia of the proximal ureter [4]. While the neoplastic potential of cystic renal lesions has been previously documented, the association between MCDK and WT is extremely reported [5].

Here, we report a case of WT arising in an MCDK, discovered incidentally through histological examination.

## Case presentation

**Figure 1:**
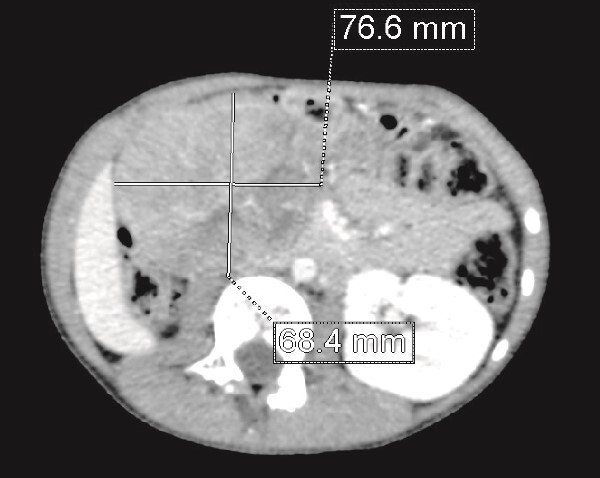
CT scan: A solid mass of 9 × 6 cm of diameter containing some areas of necrosis, well circumscribed, well encapsulated at the expense of the right kidney in contact with the cephalic portion of the pancreas and in close contact with the inferior vena cava, which is pushed to the left. The right kidney is not visualized.

**Figure 2:**
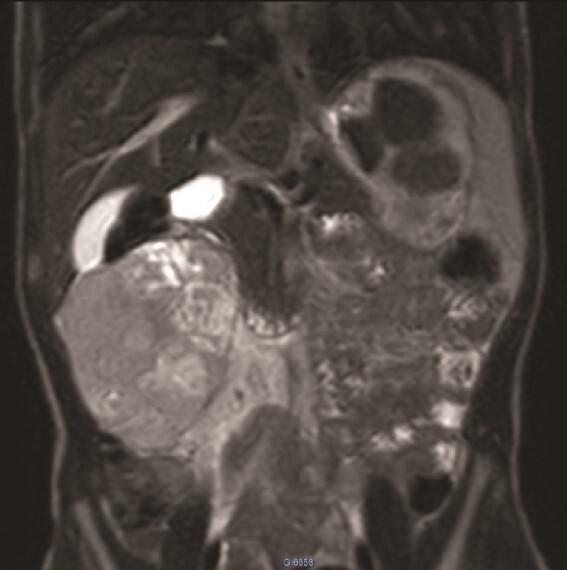
MRI: A solid mass in the upper right of the abdomen without visualization of normal right kidney parenchyma.

**Figure 3:**
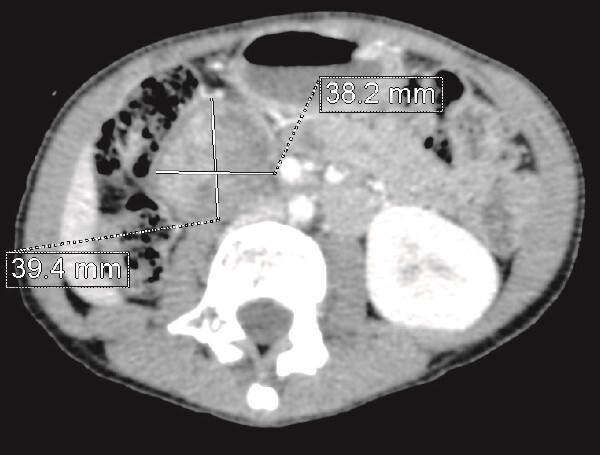
CT scan evaluation after chemotherapy: Regression in size of the right kidney mass with an estimated response of 75%.

**Figure 4:**
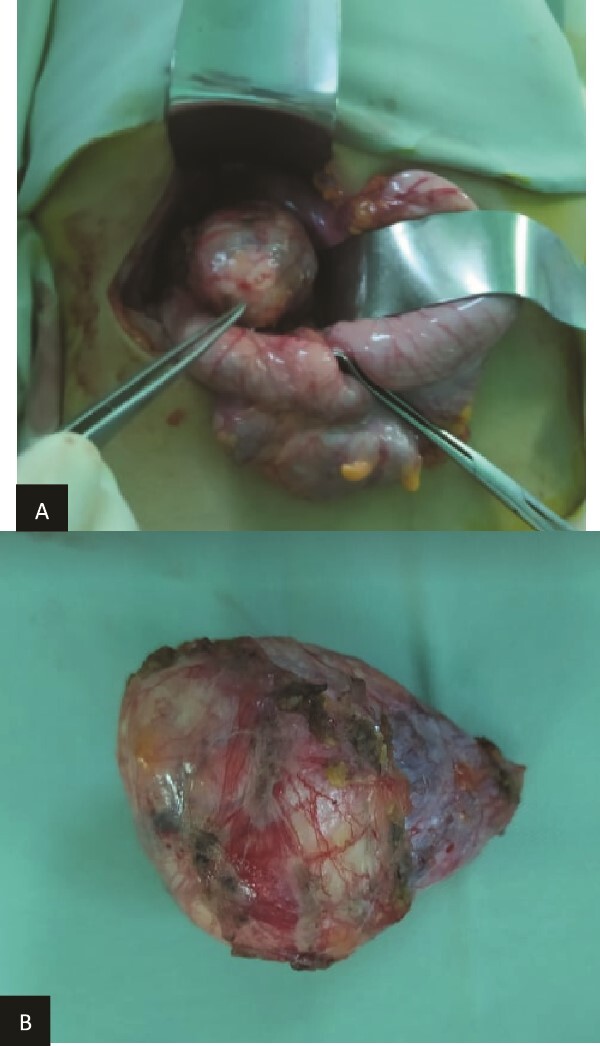
(a) Preoperatively finding. (b) Macroscopic aspect of the tumor.

We present the case of an 8-year-old boy with no significant medical history and no prenatal diagnosis of congenital renal malformation. During a physical examination following a minor abdominal injury sustained while playing, a right flank abdominal mass was discovered. Clinically, the patient’s general condition was stable. The mass was non-tender, firm, and fixed to deep planes while mobile over superficial planes. There was no evidence of varicocele. Abdominal ultrasonography and computed tomography (CT) revealed a well-circumscribed, well-encapsulated solid mass measuring 9 × 6 cm, with areas of necrosis. The mass was located in the right kidney, in contact with the cephalic portion of the pancreas and closely abutting the inferior vena cava, which was displaced to the left. The right kidney was not visualized, and no secondary lesions were detected (Fig. 1). Magnetic resonance imaging (MRI) was performed to further characterize the tumor’s origin. However, it confirmed only the presence of the tumor and the absence of normal renal parenchyma, providing no additional information regarding the origin of the upper right abdominal quadrant mass (Fig. 2). Given the unclear origin of the tumor, a CT-guided biopsy was performed. Pathological analysis of the biopsy revealed nephroblastoma without signs of anaplasia. The patient was subsequently treated with neoadjuvant chemotherapy, consisting of vincristine and actinomycin D for 4 weeks, following the SIOP 2001 protocol, and was scheduled for nephrectomy. Post-chemotherapy CT evaluation demonstrated a 75% reduction in the size of the right kidney mass (Fig. 3). The patient underwent an open nephrectomy. Intraoperatively, an atretic ureter was identified, with no visible normal renal parenchyma. An extended right nephrectomy was performed (Fig. 4a and b).

**Figure 5:**
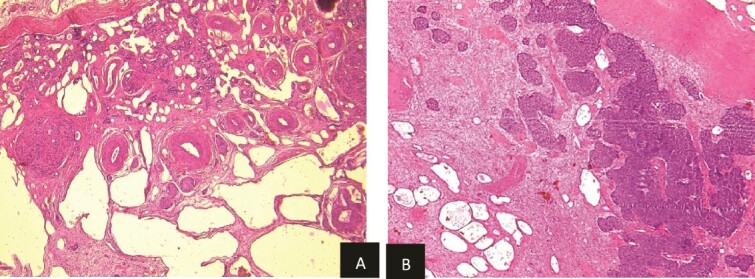
(a) Viable nephroblasma with blastemal and epithelial component (HE × 40). (b) Non-tumoral kidney tissue showing lesions of MCDK disease (HE × 40).

Histopathological examination revealed residual nephroblastoma in the right kidney post-chemotherapy. The tumor measured 5.5 cm and exhibited an estimated therapeutic response of 80%. The viable tumor consisted of an epithelial (60%) and blastemal (40%) component, with no evidence of anaplasia. A single specimen of non-tumoral renal tissue was identified in the rest of specimen, which demonstrated MCDK features, including disorganized architecture with fetal-appearing tubules and glomeruli. Some tubules were cystic and surrounded by fibro-muscular tissue, without any normal renal parenchyma (Fig. 5a and b).

Histological analysis confirmed a WT of favorable histology without capsular invasion, adjacent to an MCDK. The lesion was classified as stage I WT. The patient received a further month of dual-agent chemotherapy (vincristine and actinomycin D) without complications. Radiotherapy was not indicated. Sixteen months post-nephrectomy, the patient remains free of recurrent disease.

## Discussion

Pediatric oncology provides a unique opportunity to explore the connections between normal embryonic development, dysplasia, and neoplasia [11]. Marsden *et al.* had suggested that during normal kidney development, pluripotent metanephrogenic blastema cells become committed to forming renal tissue. While WT is usually thought to originate from committed blastema, the occasional presence of heterologous elements implies it might also arise from uncommitted blastema. Dysplastic lesions, which show metanephric features, support the idea of an origin from committed blastema, and the presence of smooth muscle does not disprove this theory [12]. The neoplastic potential of cystic lesions was described previously [13], and an association between renal cystic lesion and WT was also advanced [5]. The relationship between WT and MCDK is complex and not fully understood. While both conditions involve kidney abnormalities, evidence suggests that a direct genetic link is not established. The WT1 gene, which plays a vital role in kidney development, is strongly linked to WT and is associated with syndromes such as Denys–Drash syndrome, which elevate the risk of developing WT. However, no genetic predisposition has been directly identified connecting WT with MCDK [14]. Although some studies have reported cases of WT in patients with MCDK, these occurrences are rare and do not suggest a genetic link between the two conditions [6]. Children with MCDK are followed up throughout childhood by ultrasound because of the perceived risk of developing WT, although this risk is poorly defined and somewhat controversial. To date, in addition to our case, the association between MCDK and WT has been recognized in nine cases in the literature (Table 1). All of them were ≤2 years old [7–9, 13, 15, 16]. The present case is the first case of nephroblastoma arising in a MCDK, which was incidentally discovered on microscopic examination, diagnosed in an 8-years-old boy. The probability of malignant transformation remains a topic of debate, with many experts believing it to be extremely low or even non-existent [10]. However, although the precise prevalence of WT in children with MCDK is not well-established, some authors suggest a notable association, highlighting the importance of vigilant monitoring in these cases [17]. Additional research is necessary to better quantify and understand this relationship.

**Table 1:** Literature reports of WT associated with MCDK

Studies	Age at diagnosis	Sex	Tumor type
Andreas *et al*. [6]	2 years	Male	WT
Hartman *et al*. [7]	4 months	Female	WT
Raffensperger and Abousleiman [16]	10 months	Male	WT
Uson *et al*. [8]	3 weeks	Male	WT
Uson *et al*. [8]	9 months	Female	WT
Minevich *et al.* [9]	11 months	Male	WT
Homsy *et al.* [10]	3 months	Female	WT
Homsy *et al.* [10]	5 months	Female	WT
Present case	8 years	Male	WT

## Conclusion

The link between renal cystic dysplastic kidney lesions and WT has been documented in only few studies, with malignant transformation of blastemal cells in MCDK being even more rarely reported. Given this potential risk, it is crucial to conduct thorough evaluations for WT in patients with MCDK. To definitively establish this risk, a large cohort of children with MCDK must be followed prospectively over an extended period, continuing well into adulthood.

## Data Availability

No restriction.
